# Where Inequities Emerge: Racial and Ethnic Differences Across the COVID-19 Hospitalization Continuum

**DOI:** 10.3390/ijerph23020181

**Published:** 2026-01-31

**Authors:** Shaminul H. Shakib, Michael Goldsby, Seyed M. Karimi, Farzana Siddique, Farah N. Kanwal, Bert B. Little

**Affiliations:** 1 Department of Public Health, College of Health Sciences, Sam Houston State University, Huntsville, TX 77340, USA; 2 Department of Health Management & Systems Sciences, School of Public Health and Information Sciences, University of Louisville, Louisville, KY 40202, USA; michael.goldsby@louisville.edu (M.G.); seyed.karimi@louisville.edu (S.M.K.); bert.little@louisville.edu (B.B.L.); 3 Department of Bioinformatics & Biostatistics, School of Public Health and Information Sciences, University of Louisville, Louisville, KY 40202, USA; 4 Department of Bioengineering, J. B. Speed School of Engineering, University of Louisville, Louisville, KY 40292, USA; farzana.siddique@louisville.edu; 5 Department of Economics and Statistics, Universität Innsbruck, 6020 Innsbruck, Austria; farah.kanwal@student.uibk.ac.at

**Keywords:** COVID-19, endemic COVID-19, racial and ethnic disparities, Medicaid, hospitalization continuum, in-hospital mortality, health equity, social determinants of health, infectious disease outcomes, healthcare access

## Abstract

**Highlights:**

**Public health relevance—How does this work relate to a public health issue?**

- Racial and ethnic inequities in severe COVID-19 outcomes persisted among Medicaid beneficiaries despite uniform insurance coverage.
- Disparities were most evident at hospital admission, underscoring upstream social and structural determinants that shape exposure risk and severity at presentation.

**Public health significance—Why is this work of significance to public health?**

- By examining the hospitalization continuum (COVID-19 diagnosis at admission, excess in-hospital mortality, and mortality within COVID-19 hospitalizations), this study clarifies where inequities are most pronounced.
- Findings show that insurance coverage alone is insufficient to prevent inequities in severe infectious disease-related hospitalization burden and hospital outcomes.

**Public health implications—What are the key implications or messages for practitioners, policy makers and/or researchers in public health?**

- Equity-focused strategies should prioritize prevention, early diagnosis, and timely outpatient evaluation and treatment access to reduce avoidable hospitalizations in Medicaid and similarly vulnerable populations.
- Continuum-based evaluation of inequities can guide more targeted preparedness and health system responses for the COVID-19 endemic phase and future public health emergencies.

**Abstract:**

COVID-19 exposed longstanding racial and ethnic inequities among underserved populations. This retrospective cohort study examined inequities across stages of the hospitalization continuum—from COVID-19 diagnosis at admission to in-hospital mortality, including mortality patterns among COVID-19 hospitalizations—among Medicaid beneficiaries in Kentucky during 2020–2021. Statewide hospitalizations were analyzed using multivariable regression models, with propensity score matching (PSM) used as a confirmatory approach. Non-Hispanic Black patients were more likely than non-Hispanic White patients to be hospitalized with COVID-19 (adjusted odds ratio [aOR] = 1.41; 95% confidence interval [CI] = 1.26–1.59). Across the full cohort, COVID-19 hospitalizations were associated with substantially higher in-hospital mortality compared with non-COVID-19 hospitalizations (adjusted hazard ratio [aHR] = 2.38; 95% CI = 2.09–2.70). Additionally, hospitalizations among non-Hispanic Black patients had a modestly lower hazard of in-hospital mortality compared with non-Hispanic White patients (aHR = 0.81; 95% CI = 0.70–0.94). However, in analyses restricted to COVID-19 hospitalizations, adjusted estimates showed no Black–White differences in in-hospital mortality, with consistent findings from PSM analyses. These results indicate that racial inequities were more pronounced at hospital admission than during inpatient care, underscoring the importance of prevention, early diagnosis, and timely outpatient care as COVID-19 enters an endemic phase.

## 1. Introduction

The COVID-19 pandemic has revealed and amplified longstanding racial and ethnic health disparities in the United States. In 2021, COVID-19 was the third leading cause of death nationwide, with an unprecedented burden of mortality concentrated in communities of color [1]. Across the pandemic period from 2020 to 2021, racial and ethnic minority populations—including Black, Hispanic, and American Indian/Alaska Native (AI/AN)—experienced disproportionately high rates of COVID-19 infection, hospitalization, and death compared with White populations [2]. For example, early surveillance documented COVID-19 hospitalization rates that were approximately five times higher among Black and Hispanic persons than among White persons [3]. COVID-19 mortality rates were likewise substantially higher among Black, Hispanic, and AI/AN populations, who accounted for a disproportionately large share of deaths relative to their population size. These disparities widened with increasing age and contributed to disproportionate reductions in life expectancy among minority communities [1,3]. Taken together, these patterns reflect the unequal toll of the pandemic on structurally marginalized populations.

These inequities are rooted in structural forces that influence exposure risk, underlying health status, and access to timely care. Long-standing residential and occupational segregation, concentrated poverty, and unequal access to health services—key social determinants of health—have left communities of color disproportionately vulnerable during public health crises, including the COVID-19 pandemic [4,5]. The Centers for Disease Control and Prevention (CDC) has emphasized that these conditions are shaped by systemic and structural racism, which adversely affects health and underlies elevated risks among racial and ethnic minority populations [5]. In turn, Black and Hispanic communities were more likely to be employed in frontline and essential occupations, reside in crowded households, and experience barriers to preventive and outpatient care—conditions that increased exposure risk and delayed clinical presentation during the pandemic [6,7,8]. These structural conditions also intersect with established inequities in chronic disease burden, increasing the risk of severe illness once infected in these populations [9].

When COVID-19 infection progresses to severe illness, hospitalization represents a critical juncture at which underlying health vulnerabilities—shaped by social and economic conditions—become clinically significant. Unlike community-based outcomes, inpatient care within a standardized clinical setting involves multiple sequential processes (including diagnosis at admission, treatment, and survival) that unfold over the course of hospitalization. As a result, hospitalization provides a uniquely informative context for examining how structural inequities and baseline health risks intersect and manifest across different stages of care, rather than as a single endpoint. This hospitalization-continuum framework is especially relevant for examining health outcomes among socially and economically disadvantaged populations.

Medicaid beneficiaries represent a socially at-risk population in which these intersecting structural, social, and clinical vulnerabilities are especially concentrated. As the primary source of health coverage for low-income adults and a disproportionate share of minoritized populations, Medicaid serves individuals with elevated baseline health risks and persistent social disadvantage [10]. Kentucky provides a salient example: the state’s expanded Medicaid program covers a socioeconomically diverse population, including rural Appalachian communities with a high burden of chronic conditions [10]. Examining hospitalizations among Medicaid beneficiaries in Kentucky therefore provides an opportunity to assess how these intersecting vulnerabilities translate into inequities across the course of inpatient care.

Much of the existing research on COVID-19-related disparities has focused on single outcomes—such as infection, hospitalization, or mortality—examined in isolation [11]. Fewer studies have assessed sequential outcomes across stages of the hospitalization continuum related to COVID-19, including likelihood of diagnosis at admission, excess mortality risk, and racial and ethnic inequities in hospital mortality. To the best of our knowledge, no study to date has systematically examined these multiple stages of care within a Medicaid population. To address this gap, we analyzed a statewide retrospective cohort of Kentucky Medicaid hospitalizations during 2020–2021. This study examines racial and ethnic inequities at multiple stages of this continuum using risk-adjusted analyses, with propensity score matching serving as a confirmatory approach. Specifically, we aimed to: (1) assess racial and ethnic differences in the likelihood of COVID-19 diagnosis at hospital admission; (2) quantify excess risk of hospital mortality associated with COVID-19 compared with non-COVID-19 hospitalizations; and (3) evaluate racial and ethnic inequities in hospital mortality among COVID-19 hospitalizations. By distinguishing inequities reflected at hospital admission from those occurring during inpatient care, this study provides actionable insights to strengthen population health protection as COVID-19 enters an endemic phase.

## 2. Materials and Methods

### 2.1. Study Design

A retrospective cohort study of Kentucky Medicaid beneficiaries hospitalized during 2020–2021 was conducted to evaluate severe COVID-19 outcomes across the hospitalization continuum. The excess risk of in-hospital mortality associated with COVID-19 was estimated, and whether racial and ethnic inequities arose prior to hospitalization or persisted after admission was assessed. The study was reviewed by the University of Louisville Institutional Review Board (IRB 23.0537), determined to be non-human subjects research, and was reported in accordance with the Strengthening the Reporting of Observational Studies in Epidemiology (STROBE) guidelines [12].

### 2.2. Data Source

Data were obtained from the Kentucky Health Facility and Services (KHFS) claims database for calendar years 2020–2021, maintained by the Cabinet for Health and Family Services [13]. The KHFS database contains encounter-level inpatient discharge records from hospitals statewide in Kentucky, including patient demographics, primary payer, admission and discharge details, hospital characteristics, and International Classification of Diseases, Tenth Revision (ICD-10) diagnosis and procedure codes. The unit of analysis was hospitalization. Because the data were de-identified and did not include unique patient identifiers, repeated admissions could not be linked and were therefore treated as independent encounters.

### 2.3. Study Sample

**Figure 1:**
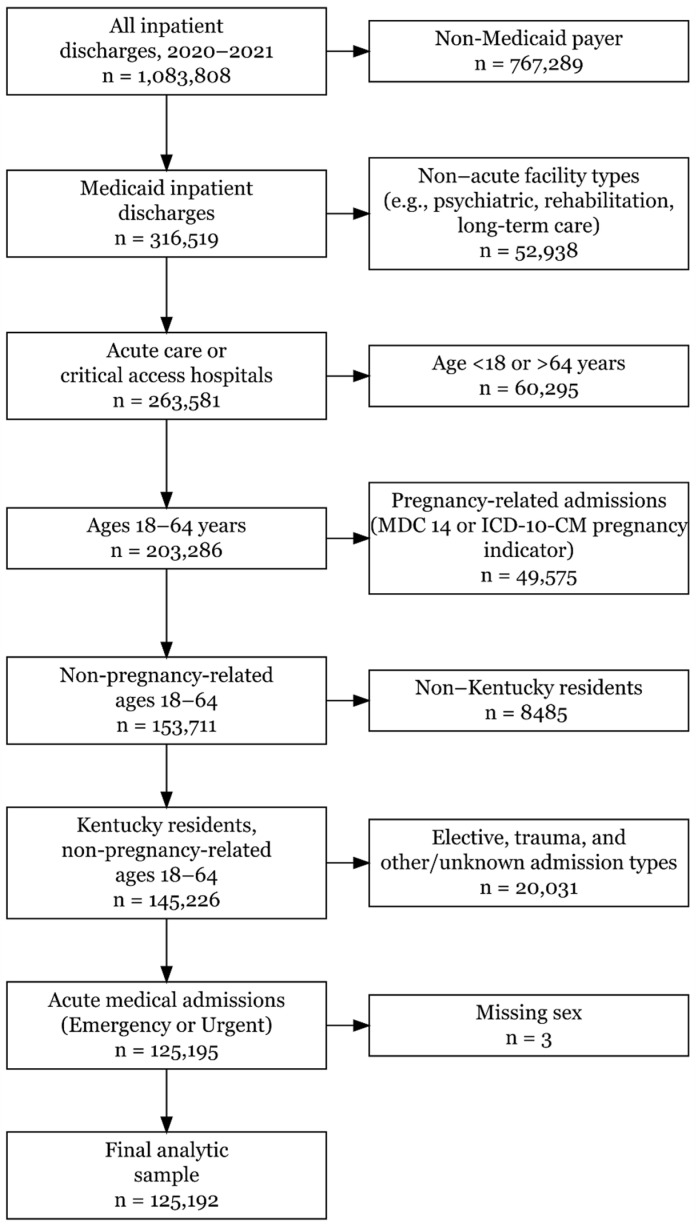
Flowchart illustrating the inclusion and exclusion criteria used to derive the final analytic cohort (n = 125,192) from all Kentucky inpatient discharges, 2020–2021.

The study sample was derived using sequential inclusion and exclusion criteria, as illustrated in Figure 1.

Hospitalizations were restricted to Medicaid beneficiaries aged 18–64 years who were Kentucky residents and admitted for non-pregnancy-related, acute medical conditions. Pregnancy-related admissions were identified using Major Diagnostic Category (MDC) 14 and ICD-10-CM pregnancy diagnosis codes and excluded from the analytic sample. The analytic cohort was limited to admissions from short-term acute care hospitals and critical access hospitals; admissions from other facility types, elective or trauma-related admissions, and those with missing demographic information were excluded. After these criteria were applied, the final analytic sample comprised 125,192 Medicaid inpatient hospitalizations.

### 2.4. Outcome and Exposure

Two primary outcomes were examined: COVID-19 diagnosis at hospitalization and in-hospital mortality. Hospitalization with a COVID-19 diagnosis was defined using ICD-10-CM diagnosis codes B97.29 and/or U07.1 [14]. In-hospital mortality was identified using the discharge disposition code indicating death during the hospitalization (code 20). Across analyses, this hospitalization-level COVID-19 diagnosis indicator was modeled as the outcome, the primary exposure, or used to restrict the analytic sample, as appropriate.

### 2.5. Covariates

All multivariable analyses adjusted for patient- and hospital-level characteristics selected a priori based on existing literature and data availability. Continuous variables were categorized where appropriate to increase interpretability and consistency with prior health services and COVID-19 outcomes research. Patient-level covariates included age, sex, and comorbidity burden measured using the Charlson Comorbidity Index (CCI). The CCI score was calculated for each hospitalization using ICD-10-CM diagnosis codes according to a validated coding algorithm and categorized for analysis [15].

Hospital-level covariates included hospital type (acute care vs. critical access), bed size, and ownership. Calendar time was accounted for using discharge quarter to adjust for temporal variation in COVID-19 burden, treatment availability, and hospital capacity during the study period.

### 2.6. Statistical Analysis

Descriptive statistics were produced to summarize patient and hospital characteristics for hospitalizations with and without a COVID-19 diagnosis. Categorical variables were summarized using frequencies and percentages, and length of stay (LOS) in days was summarized using medians and interquartile ranges (IQRs).

To evaluate racial and ethnic inequities across the hospitalization continuum and excess risk of in-hospital mortality associated with COVID-19, a series of multivariable regression analyses were conducted. A logistic regression model was estimated to assess the odds of COVID-19 diagnosis at hospitalization. Race/ethnicity was specified as the primary explanatory variable, with adjustment for patient- and hospital-level covariates. Odds ratios (ORs) and 95% confidence intervals (CIs) were reported.

Unadjusted in-hospital survival by COVID-19 diagnosis was described using Kaplan–Meier survival curves, with discharge alive treated as a censoring event. Excess in-hospital mortality associated with COVID-19 diagnosis was quantified using Cox proportional hazards regression models, with LOS as the time scale and death during hospitalization as the event. Hazard ratios (HRs) and 95% CIs were reported.

To evaluate whether racial inequities in in-hospital mortality persisted among hospitalizations with a COVID-19 diagnosis, analyses were restricted to this subset, and Cox proportional hazards models analogous to those described above were estimated.

In a confirmatory analysis, propensity score matching (PSM) was used to create covariate-balanced, comparable groups of hospitalizations among non-Hispanic (NH) Black and NH White individuals with a COVID-19 diagnosis. Matching was performed on patient- and hospital-level covariates using nearest-neighbor 1:1 matching without replacement and a caliper of 0.05 on the propensity score. Cox proportional hazards regression was then repeated in the matched sample to confirm findings on in-hospital mortality.

In all regression models, robust standard errors clustered at the hospital level were used to account for correlation among hospitalizations within the same facility. Proportional hazards assumptions for Cox models were assessed using standard diagnostic methods, including log–log survival plots and Schoenfeld residuals, with no substantive violations observed. This was a hypothesis-driven comparative analysis; therefore, multiple testing corrections were not applied to avoid overly conservative inference. The data were managed in KNIME (version 5.1) and analyzed in Stata (version 18). Statistical significance was assessed using two-sided tests with α = 0.05.

### 2.7. Sensitivity Analyses

Several sensitivity analyses were conducted to assess the robustness of the primary modeling assumptions and findings. First, Cox proportional hazards models for in-hospital mortality were repeated after excluding hospitalizations with LOS shorter than 1 day or longer than 60 days, which may reflect atypical clinical courses or administrative irregularities.

Second, comorbidity specification was evaluated by replacing categorical CCI measures with the continuous CCI score in both logistic regression and Cox proportional hazards models. Third, an alternative propensity score-matched sample of hospitalizations with a COVID-19 diagnosis was constructed using 1:2 nearest-neighbor matching without replacement and a caliper of 0.10, and a Cox proportional hazards model was re-estimated to assess sensitivity to the matching ratio and caliper width.

All sensitivity analyses used the same covariates as their corresponding primary models. Detailed results, including full Cox model estimates for hospitalizations with a COVID-19 diagnosis, propensity score matching diagnostics from the 1:1 matched analysis, and additional sensitivity analyses, are provided in the Appendix A.

## 3. Results

### 3.1. Study Sample and Cohort Derivation

After applying sequential inclusion and exclusion criteria (Figure 1), 125,192 hospitalizations among Medicaid-enrolled Kentucky residents aged 18–64 years during 2020–2021 comprised the analytic cohort.

### 3.2. Descriptive Characteristics by COVID-19 Diagnosis

Among the 125,192 hospitalizations, 8409 (6.7%) had a COVID-19 diagnosis and 116,783 (93.3%) did not (Table 1). Hospitalizations with a COVID-19 diagnosis were more concentrated in older age groups; individuals aged 60–64 years accounted for 1492 (17.7%) COVID-19 hospitalizations compared with 15,731 (13.5%) non-COVID-19 hospitalizations. Sex distributions were similar across groups, with females comprising 4285 (51.0%) COVID-19 hospitalizations and 57,393 (49.1%) non-COVID-19 hospitalizations.

Overall, hospitalizations among non-Hispanic White patients comprised the majority of encounters. Among Hispanic patients, COVID-19 hospitalizations accounted for a larger share (556; 6.6%) than non-COVID-19 hospitalizations (2452; 2.1%), whereas the proportions of hospitalizations among non-Hispanic Black patients were similar across COVID-19 (1253; 14.9%) and non-COVID-19 hospitalizations (15,189; 13.0%).

Median length of stay was longer for COVID-19 hospitalizations (5 days; IQR, 3–9) than for non-COVID-19 hospitalizations (3 days; IQR, 2–6). The distribution of Charlson Comorbidity Index (CCI) categories differed between COVID-19 and non-COVID-19 hospitalizations; a smaller share of COVID-19 hospitalizations fell into the highest category (≥5: 1941; 23.1%) compared with non-COVID-19 hospitalizations (≥5: 34,403; 29.5%).

COVID-19 hospitalizations occurred more frequently in critical access hospitals (3215; 38.2%) than non-COVID-19 hospitalizations (40,545; 34.7%). COVID-19 hospitalizations were also concentrated in larger hospital bed-size categories and varied across discharge quarters, with peaks in 2020 Q4, 2021 Q1, and 2021 Q3.

### 3.3. Adjusted Odds of COVID-19 Diagnosis at Hospitalization

In multivariable logistic regression analysis, race and ethnicity were associated with the odds of COVID-19 diagnosis at hospitalization (Table 2). Compared with hospitalizations among non-Hispanic White patients, higher adjusted odds of COVID-19 diagnosis were observed for hospitalizations among non-Hispanic Black (aOR = 1.41; 95% CI, 1.26–1.59), Hispanic (aOR = 4.14; 95% CI, 3.38–5.08), non-Hispanic Asian (aOR = 2.59; 95% CI, 1.98–3.38), and non-Hispanic Native Hawaiian or Pacific Islander patients (aOR = 4.07; 95% CI, 2.52–6.57). Increasing age was associated with higher adjusted odds of COVID-19 diagnosis relative to ages 18–20, with the highest odds among hospitalizations of patients aged 60–64 years (aOR = 2.91; 95% CI, 2.29–3.69). Hospitalizations among female patients had slightly higher adjusted odds of COVID-19 diagnosis than those among male patients (aOR = 1.10; 95% CI, 1.05–1.16). Adjusted odds varied across discharge quarters, with elevated odds observed during late 2020 and throughout 2021.

### 3.4. Unadjusted Survival by COVID-19 Diagnosis

**Figure 2:**
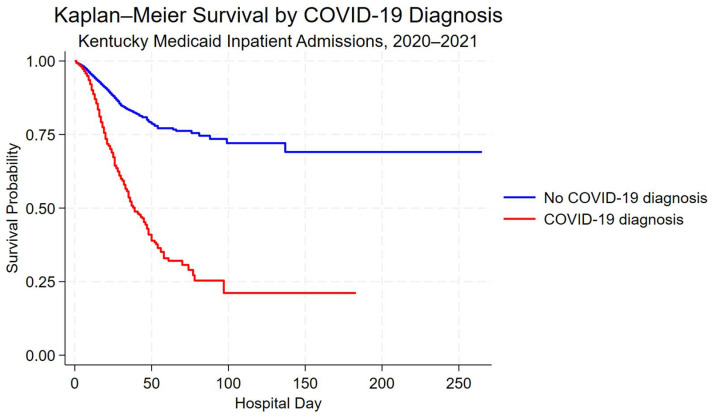
Kaplan–Meier survival curves for hospitalizations with and without a COVID-19 diagnosis among Kentucky Medicaid beneficiaries, 2020–2021. Survival time is measured in-hospital days; discharge alive was treated as a censoring event.

Kaplan–Meier survival curves demonstrated lower unadjusted in-hospital survival among hospitalizations with a COVID-19 diagnosis compared with those without a COVID-19 diagnosis (Figure 2). Differences in survival probability were most pronounced during the first 60 days of hospitalization.

### 3.5. Adjusted In-Hospital Mortality: COVID-19 vs. Non-COVID-19 Hospitalizations

In adjusted Cox proportional hazards models, COVID-19 diagnosis was associated with a higher hazard of in-hospital mortality compared with non-COVID-19 hospitalizations (aHR = 2.38; 95% CI, 2.09–2.70) (Table 3). Increasing age was associated with progressively higher hazards of mortality relative to ages 18–20, with the highest hazards observed among hospitalizations of patients aged 55–59 years (aHR = 1.88; 95% CI, 1.16–3.05) and 60–64 years (aHR = 2.15; 95% CI, 1.34–3.45).

Compared with hospitalizations among non-Hispanic White patients, hospitalizations among non-Hispanic Black patients had a lower adjusted hazard of in-hospital mortality (aHR = 0.81; 95% CI, 0.70–0.94). Higher CCI categories were associated with increasing mortality hazards, with the highest hazard observed among hospitalizations with CCI ≥5 (aHR = 2.61; 95% CI, 2.01–3.38).

### 3.6. Racial Inequities in In-Hospital Mortality Among COVID-19 Hospitalizations

Among hospitalizations with a COVID-19 diagnosis (n = 7757; deaths = 650), adjusted Cox regression models showed no difference in in-hospital mortality between non-Hispanic Black and non-Hispanic White patients (aHR = 0.89; 95% CI, 0.70–1.13; Table 4). Full model estimates are provided in Appendix A Table A1.

In confirmatory analyses using 1:1 propensity score-matched samples of hospitalizations with a COVID-19 diagnosis among non-Hispanic Black and non-Hispanic White patients (n = 1889; deaths = 114), the adjusted hazard of in-hospital mortality did not differ between groups (aHR = 1.10; 95% CI, 0.69–1.74) (Table 4). Covariate balance improved after matching, with standardized differences below 0.10 for most covariates (Appendix A Table A2).

### 3.7. Sensitivity Analyses

Results were robust across sensitivity analyses (Appendix A Table A3, Table A4 and Table A5). Excluding hospitalizations with length of stay shorter than 1 day or longer than 60 days yielded similar hazard estimates for in-hospital mortality among hospitalizations with and without a COVID-19 diagnosis (HR = 2.35; 95% CI, 2.08–2.65), as well as for hospitalizations among non-Hispanic Black versus non-Hispanic White patients with a COVID-19 diagnosis (HR = 0.90; 95% CI, 0.71–1.14). Replacing categorical CCI measures with a continuous CCI score produced comparable estimates for COVID-19 diagnosis at hospitalization and in-hospital mortality. Alternative propensity score matching using 1:2 nearest-neighbor matching also yielded similar mortality hazard estimates (HR = 0.86; 95% CI, 0.63–1.17).

## 4. Discussion

In this statewide analysis of hospitalizations among Medicaid beneficiaries in Kentucky during the COVID-19 pandemic (2020–2021), racial and ethnic inequities were observed across multiple stages of the hospitalization continuum. Compared with non-Hispanic White patients, non-Hispanic Black patients were more likely to be hospitalized with COVID-19 rather than other conditions, indicating a disproportionate burden at the point of hospital admission. In the full cohort model, hospitalizations involving a COVID-19 diagnosis were associated with substantially higher in-hospital mortality compared with non-COVID-19 hospitalizations. Further, in-hospital mortality risk was modestly lower among non-Hispanic Black patients than among non-Hispanic White patients—a pattern likely reflecting differences in case mix or admission thresholds rather than COVID-19-specific mortality differences.

When analyses were restricted to COVID-19 hospitalizations, adjusted models did not show Black–White differences in in-hospital mortality. Together, these findings suggest that racial inequities were more pronounced at the point of hospitalization and in the distribution of COVID-19 admissions, not within COVID-19-specific inpatient mortality. This distinction underscores the importance of examining multiple stages of hospitalization when assessing racial and ethnic inequities.

For instance, the distribution of COVID-19 hospitalizations by race and ethnicity in this study mirrored patterns observed nationally during the pandemic [16,17]. Greater exposure risk stemming from structural inequities—including frontline and essential occupations, crowded living conditions, and barriers to timely outpatient care—likely contributed to higher infection risk and subsequent hospitalization among non-Hispanic Black and Hispanic communities [18]. National surveillance has repeatedly documented substantially higher COVID-19 hospitalization rates among non-Hispanic Black, American Indian/Alaska Native, and Hispanic populations compared with non-Hispanic White populations during the early pandemic period [16,17].

Importantly, within Kentucky’s Medicaid population, these patterns persisted despite uniform insurance coverage, suggesting that coverage alone was insufficient to mitigate disparities in severe COVID-19 illness requiring hospitalization. This underscores the role of upstream population- and environmental-level factors—such as occupational exposures, housing conditions, structural barriers to care, comorbidity burden, and healthcare-seeking patterns—in shaping which patients arrive at the hospital with COVID-19.

Beyond differences in who was hospitalized with COVID-19, outcomes during inpatient care further highlight the burden associated with COVID-19 hospitalizations. Hospitalizations involving a COVID-19 diagnosis were associated with a substantially higher risk of in-hospital mortality compared with non-COVID-19 hospitalizations. This elevated mortality persisted after adjustment for demographic characteristics and comorbidity burden, indicating that differences in patient case mix alone do not fully explain the observed mortality gap. Consistent with these findings, national evidence from the early pandemic period documented markedly higher in-hospital death rates among patients hospitalized with COVID-19 compared with other acute conditions [19].

From a public health perspective, these findings underscore that preventing COVID-19 hospitalizations among high-risk populations remains critical, even as the pandemic transitions to an endemic phase. The markedly higher mortality associated with COVID-19 once inpatient care is required highlights the importance of timely access to vaccination, workplace protections (e.g., paid sick leave, PPE, safer conditions), early outpatient evaluation, and effective antiviral treatment—particularly for Medicaid populations facing structural barriers to care [19,20]. Strengthening these upstream and early-care interventions may reduce the disproportionate burden of severe disease that ultimately manifests within hospitals [21].

Several large multisite studies have found that, after adjustment for demographic and clinical factors, racial differences in COVID-19 in-hospital mortality are often attenuated or not statistically significant, even as disparities in infection and hospitalization persist [22,23]. Consistent with this evidence, adjusted analyses restricted to hospitalizations with a COVID-19 diagnosis did not demonstrate differences in in-hospital mortality between non-Hispanic Black and non-Hispanic White patients. This contrast suggests that inequities in this Medicaid population were more strongly reflected in who arrived at the hospital with COVID-19 than in mortality once hospitalized.

Notably, the absence of observed mortality differences within COVID-19 hospitalizations should not be interpreted as evidence that inpatient care processes were equivalent across racial groups. Residual differences in illness severity at presentation, unmeasured clinical factors, and social conditions preceding admission may continue to influence outcomes in ways not fully captured by administrative data. Confirmatory propensity score-matched analyses among COVID-19 hospitalizations yielded consistent results, supporting the interpretation that disparities in this cohort were concentrated earlier in the hospitalization continuum rather than during inpatient mortality.

Taken together, the patterns observed here highlight why evaluating multiple points along the hospitalization continuum is essential for identifying where inequities are most pronounced and where interventions may be most effective.

## 5. Strengths and Limitations

This study has several strengths. First, a statewide cohort of Medicaid hospitalizations in Kentucky was analyzed across 2020–2021. The dataset captures inpatient encounters within a single public insurance program, enabling assessment of inequities within a socioeconomically disadvantaged population. This comprehensive coverage reduces potential selection bias related to insurance status and supports stable estimates within the Medicaid population.

Second, inequities were examined across multiple stages of the hospitalization continuum. These stages included likelihood of COVID-19 diagnosis at admission, excess in-hospital mortality associated with COVID-19, and mortality patterns among COVID-19 hospitalizations. This continuum-based approach extends prior studies that often focused on single outcomes in isolation and helps clarify where inequities are most pronounced [11].

Third, rigorous analytic strategies were used to strengthen robustness. Multivariable regression models adjusted for a wide range of demographic and clinical factors, and propensity score matching was used as a confirmatory approach for key comparisons. Consistent findings across analytic approaches increase confidence in the robustness of the observed associations, while acknowledging the non-causal nature of the study. Finally, the use of administrative claims data enabled population-level analyses that are difficult to achieve in smaller clinical cohorts.

Several limitations should be considered. First, as an observational retrospective study, the analyses cannot establish causal relationships between race/ethnicity, COVID-19 diagnosis, and in-hospital mortality. Second, although models adjusted for a wide range of covariates, residual confounding remains possible. Because this study relied on administrative claims data, direct measures of illness severity at presentation (e.g., physiologic indicators, laboratory values, or radiographic findings) were unavailable and may influence in-hospital mortality. In addition, diagnoses, comorbidities, and race/ethnicity were derived from administrative records and may be misclassified, and claims data may not fully capture clinical nuance or key social risk factors relevant to COVID-19 outcomes.

Third, hospitalizations—not individual patients—were the unit of analysis. Repeating admissions by the same individual could contribute multiple observations and may influence estimates despite adjustment. Differences in the severity of illness at the time of hospital admission across racial and ethnic groups may contribute to selection bias, particularly when examining disparities at the point of hospitalization.

Fourth, race and ethnicity categories were constrained by data availability and aggregation requirements. More granular subgroups (e.g., Hispanic origin groups) could not be examined, and small sample sizes limited precision for some populations, including Asian and American Indian/Alaska Native patients. Fifth, the study period reflects the early pandemic (2020–2021), before widespread vaccine uptake and the introduction of newer COVID-19 therapies. Patterns may have evolved later, although the structural drivers of inequity emphasized here remain relevant for preparedness and future public health emergencies.

Finally, because the analysis was limited to Medicaid beneficiaries in Kentucky, findings may not be fully generalizable to other populations or geographic settings. Future studies incorporating patient-level longitudinal data, clinical severity measures, and broader geographic coverage may further clarify the mechanisms underlying observed disparities.

## 6. Conclusions

This study demonstrates that racial and ethnic inequities in COVID-19 outcomes among Medicaid beneficiaries were concentrated at the point of hospitalization rather than within COVID-19 in-hospital mortality. Non-Hispanic Black Medicaid patients bore a disproportionate burden of COVID-19 hospital admissions, consistent with upstream structural and social conditions that shape exposure risk and baseline health prior to hospitalization.

These findings highlight the importance of distinguishing inequities reflected at hospital entry from those occurring during inpatient care. As COVID-19 enters an endemic phase and severe outcomes become increasingly concentrated among socioeconomically vulnerable populations, equitable response will depend on prevention, early diagnosis, and timely access to outpatient treatment. Medicaid programs and public health systems remain central to reducing avoidable hospitalizations by addressing risks that precede hospital entry.

Evaluating outcomes across multiple stages of hospitalization clarifies where inequities arise and where interventions may be most effective. Preparedness strategies that strengthen early prevention and equitable access to care—particularly for Medicaid populations—will be essential to reducing disparities in severe disease during COVID-19 and future public health emergencies.

## Figures and Tables

**Table 1:** Characteristics of Hospitalizations Among Medicaid Patients by COVID-19 Status, Kentucky, 2020–2021.

	Overall (n = 125,192)	Non-COVID-19 (n = 116,783)	COVID-19 (n = 8409)
	n (%) ^1^	n (%) ^1^	n (%) ^1^
Age Group			
18–20	3118 (2.49%)	2988 (2.56%)	130 (1.55%)
21–24	4895 (3.91%)	4666 (4.00%)	229 (2.72%)
25–29	9168 (7.32%)	8673 (7.43%)	495 (5.89%)
30–34	11,485 (9.17%)	10,870 (9.31%)	615 (7.31%)
35–39	12,914 (10.32%)	12,141 (10.40%)	773 (9.19%)
40–44	14,899 (11.90%)	13,981 (11.97%)	918 (10.92%)
45–49	15,000 (11.98%)	13,985 (11.98%)	1015 (12.07%)
50–54	17,656 (14.10%)	16,342 (13.99%)	1314 (15.63%)
55–59	18,834 (15.04%)	17,406 (14.90%)	1428 (16.98%)
60–64	17,223 (13.76%)	15,731 (13.47%)	1492 (17.74%)
Sex			
Male	63,514 (50.73%)	59,390 (50.86%)	4124 (49.04%)
Female	61,678 (49.27%)	57,393 (49.14%)	4285 (50.96%)
Race/Ethnicity ^2^			
NH White	104,988 (83.86%)	98,484 (84.33%)	6504 (77.35%)
NH Black	16,442 (13.13%)	15,189 (13.01%)	1253 (14.90%)
NH Asian	553 (0.44%)	477 (0.41%)	76 (0.90%)
NH AI/AN	122 (0.10%)	116 (0.10%)	6 (0.07%)
NH NH/PI	79 (0.06%)	65 (0.06%)	14 (0.17%)
Hispanic	3008 (2.40%)	2452 (2.10%)	556 (6.61%)
Length of stay, days, median (IQR) ^3^	-	3 (2–6)	5 (3–9)
CCI Score ^4^			
0	38,788 (30.98%)	36,074 (30.89%)	2714 (32.27%)
1–2	34,375 (27.46%)	31,725 (27.17%)	2650 (31.51%)
3–4	15,685 (12.53%)	14,581 (12.49%)	1104 (13.13%)
≥5	36,344 (29.03%)	34,403 (29.46%)	1941 (23.08%)
Hospital Type			
Critical Access ^5^	43,760 (34.95%)	40,545 (34.72%)	3215 (38.23%)
Acute Care	81,432 (65.05%)	76,238 (65.28%)	5194 (61.77%)
Hospital Bed Size			
Under 1000 beds	1694 (1.35%)	1494 (1.28%)	200 (2.38%)
1000–2999 beds	8577 (6.85%)	7845 (6.72%)	732 (8.70%)
3000–5999 beds	16,947 (13.54%)	15,760 (13.50%)	1187 (14.12%)
6000–9999 beds	19,498 (15.57%)	18,080 (15.48%)	1418 (16.86%)
Over 9999 beds	78,476 (62.68%)	73,604 (63.03%)	4872 (57.94%)
Hospital Ownership			
Non-Profit	111,777 (89.28%)	104,166 (89.20%)	7611 (90.51%)
For-Profit	13,415 (10.72%)	12,617 (10.80%)	798 (9.49%)
Discharge Quarter			
2020 Q1	14,913 (11.91%)	14,869 (12.73%)	44 (0.52%)
2020 Q2	15,033 (12.01%)	14,633 (12.53%)	400 (4.76%)
2020 Q3	16,461 (13.15%)	15,929 (13.64%)	532 (6.33%)
2020 Q4	14,964 (11.95%)	13,673 (11.71%)	1291 (15.35%)
2021 Q1	15,644 (12.50%)	14,338 (12.28%)	1306 (15.53%)
2021 Q2	16,413 (13.11%)	15,865 (13.59%)	548 (6.52%)
2021 Q3	16,156 (12.90%)	13,618 (11.66%)	2538 (30.18%)
2021 Q4	15,608 (12.47%)	13,858 (11.87%)	1750 (20.81%)

^1^ Values are presented as n (%) unless otherwise indicated. Percentages may not sum to 100 because of rounding. ^2^ Race/ethnicity categories are mutually exclusive. NH = Non-Hispanic; AI/AN = American Indian or Alaska Native; NH/PI = Native Hawaiian or Pacific Islander. ^3^ Length of stay is reported as median (interquartile range). ^4^ CCI = Charlson Comorbidity Index. ^5^ Critical Access Hospitals (CAHs) are CMS-designated rural facilities with limited inpatient capacity and 24-h emergency care.

**Table 2:** Adjusted Odds of COVID-19 Diagnosis at Hospitalization Among Medicaid Beneficiaries, Kentucky, 2020–2021.

	Adjusted OR ^1^	95% CI ^2^		*p*-Value
		Lower	Upper	
Age Group (ref = 18–20)				
21–24	1.18	0.9	1.56	0.237
25–29	1.43	1.11	1.83	0.005
30–34	1.42	1.13	1.77	0.002
35–39	1.64	1.31	2.05	<0.001
40–44	1.72	1.37	2.17	<0.001
45–49	2.01	1.6	2.53	<0.001
50–54	2.34	1.86	2.93	<0.001
55–59	2.46	1.95	3.12	<0.001
60–64	2.91	2.29	3.69	<0.001
Sex (ref = Male)				
Female	1.1	1.05	1.16	<0.001
Race/Ethnicity (ref = NH White) ^3^				
NH Black	1.41	1.26	1.59	<0.001
NH Asian	2.59	1.98	3.38	<0.001
NH AI/AN	0.76	0.34	1.69	0.501
NH NH/PI	4.07	2.52	6.57	<0.001
Hispanic	4.14	3.38	5.08	<0.001
CCI Score (ref = 0) ^4^				
1–2	0.98	0.88	1.09	0.705
3–4	0.82	0.72	0.94	0.004
≥5	0.59	0.52	0.68	<0.001
Hospital Type (ref = Critical Access) ^5^				
Acute Care	0.86	0.67	1.1	0.235
Hospital Bed Size (ref = <1000 beds)				
1000–2999 beds	0.97	0.73	1.28	0.821
3000–5999 beds	0.73	0.55	0.96	0.026
6000–9999 beds	0.71	0.44	1.14	0.152
Over 9999 beds	0.61	0.46	0.82	0.001
Hospital Ownership (ref = Non-Profit)				
For-Profit	0.71	0.54	0.94	0.017
Discharge Quarter (ref = 2020 Q1)				
2020 Q2	8.99	5.5	14.68	<0.001
2020 Q3	11.13	8.1	15.3	<0.001
2020 Q4	31.28	22.04	44.4	<0.001
2021 Q1	30.53	21.39	43.57	<0.001
2021 Q2	11.42	8.12	16.05	<0.001
2021 Q3	62.57	41.94	93.35	<0.001
2021 Q4	41.76	28.62	60.94	<0.001

^1^ OR = odds ratio. ^2^ CI = confidence interval. ^3^ Race/ethnicity categories are mutually exclusive. NH = Non-Hispanic; AI/AN = American Indian or Alaska Native; NH/PI = Native Hawaiian or Pacific Islander. ^4^ CCI = Charlson Comorbidity Index. ^5^ Critical Access Hospitals (CAHs) are CMS-designated rural facilities with limited inpatient capacity and 24-h emergency care.

**Table 3:** Adjusted Hazard of Inpatient Mortality for COVID-19 vs. Non-COVID-19 Hospitalizations Among Medicaid Patients, Kentucky, 2020–2021.

	Adjusted HR ^1^	95% CI ^2^		*p*-Value
		Lower	Upper	
Exposure (ref = Non-COVID-19)				
COVID-19	2.38	2.09	2.7	<0.001
Age Group (ref = 18–20)				
21–24	0.87	0.6	1.25	0.449
25–29	1.03	0.72	1.47	0.888
30–34	1.35	0.88	2.07	0.171
35–39	1.39	0.86	2.25	0.177
40–44	1.43	0.92	2.21	0.11
45–49	1.62	1.06	2.48	0.026
50–54	1.62	1.00	2.63	0.052
55–59	1.88	1.16	3.05	0.011
60–64	2.15	1.34	3.45	0.001
Sex (ref = Male)				
Female	0.90	0.84	0.97	0.006
Race/Ethnicity (ref = NH White) ^3^				
NH Black	0.81	0.70	0.94	0.006
NH Asian	0.79	0.49	1.29	0.348
NH AI/AN	0.94	0.33	2.69	0.903
NH NH/PI	1.02	0.34	3.07	0.969
Hispanic	0.90	0.72	1.12	0.339
CCI Score (ref = 0) ^4^				
1–2	1.45	1.17	1.81	0.001
3–4	1.59	1.20	2.10	0.001
≥5	2.61	2.01	3.38	<0.001
Hospital Type (ref = Critical Access) ^5^				
Acute Care	0.92	0.71	1.19	0.519
Hospital Bed Size (ref = <1000 beds)				
1000–2999 beds	0.78	0.42	1.45	0.435
3000–5999 beds	0.98	0.55	1.77	0.957
6000–9999 beds	0.95	0.51	1.79	0.883
Over 9999 beds	1.19	0.67	2.13	0.554
Hospital Ownership (ref = Non-Profit)				
For-Profit	0.94	0.75	1.19	0.609
Discharge Quarter (ref = 2020 Q1)				
2020 Q2	1.24	1.04	1.47	0.018
2020 Q3	1.16	1.00	1.35	0.05
2020 Q4	1.19	1.02	1.40	0.025
2021 Q1	1.02	0.90	1.16	0.759
2021 Q2	1.11	0.96	1.3	0.162
2021 Q3	1.40	1.20	1.63	<0.001
2021 Q4	1.27	1.09	1.48	0.002

^1^ HR = Hazard ratio. ^2^ CI = confidence interval. ^3^ Race/ethnicity categories are mutually exclusive. NH = Non-Hispanic; AI/AN = American Indian or Alaska Native; NH/PI = Native Hawaiian or Pacific Islander. ^4^ CCI = Charlson Comorbidity Index. ^5^ Critical Access Hospitals (CAHs) are CMS-designated rural facilities with limited inpatient capacity and 24-h emergency care.

**Table 4:** Black–White Differences in In-Hospital Mortality Among Medicaid Patients Hospitalized With COVID-19, Kentucky, 2020–2021.

	Adjusted HR ^1^	95% CI ^2^		*p*-Value
		Lower	Upper	
Adjusted Cox Model ^3^ (n = 7757; deaths = 650)				
NH Black (ref = NH White) ^4^	0.89	0.70	1.13	0.326
Propensity Score-Matched (PSM) Cox Model ^5^ (n = 1889; deaths = 114)				
NH Black (ref = NH White) ^4^	1.10	0.69	1.74	0.692

^1^ HR = hazard ratio. ^2^ CI = confidence interval. ^3^ Adjusted for age group, sex, CCI scores, hospital characteristics, and discharge quarter. ^4^ NH = non-Hispanic; NH Black and NH White are mutually exclusive race/ethnicity categories. ^5^ PSM restricted to NH Black and NH White COVID-19-positive inpatients; matched 1:1 on demographics, CCI scores, hospital characteristics, and discharge quarter using nearest-neighbor matching (caliper 0.05).

## Data Availability

Kentucky Health Facility and Services (KHFS) inpatient data was used to identify Medicaid patients. This data can be requested from the Commonwealth Institute of Kentucky, University of Louisville.

## Appendix A

**Table A1:** Full Multivariable Cox Proportional Hazards Model for In-Hospital Mortality Among COVID-19-Positive Medicaid Patients, Kentucky, 2020–2021 (n = 7757; deaths = 650).

	Adjusted HR ^1^	95% CI ^2^		*p*-Value
		Lower	Upper	
Age Group (ref = 18–20)				
21–24	1.29	0.41	4.00	0.664
25–29	0.94	0.32	2.79	0.911
30–34	1.86	0.59	5.84	0.289
35–39	1.44	0.47	4.42	0.522
40–44	2.15	0.74	6.27	0.162
45–49	2.25	0.77	6.59	0.141
50–54	2.38	0.78	7.26	0.129
55–59	2.57	0.86	7.70	0.093
60–64	2.92	0.96	8.86	0.058
Sex (ref = Male)				
Female	0.81	0.68	0.95	0.009
Race/Ethnicity (ref = NH White) ^3^				
NH Black	0.89	0.70	1.13	0.326
NH Asian	0.84	0.34	2.12	0.717
NH AI/AN	1.58	0.10	24.52	0.744
NH NH/PI	0.93	0.23	3.69	0.914
Hispanic	0.91	0.68	1.21	0.507
CCI Score (ref = 0) ^4^				
1–2	1.28	1.04	1.58	0.018
3–4	1.06	0.81	1.40	0.652
≥5	1.40	1.09	1.79	0.008
Hospital Type (ref = Critical Access) ^5^				
Acute Care	0.72	0.58	0.90	0.004
Hospital Bed Size (ref = <1000 beds)				
1000–2999 beds	0.84	0.43	1.64	0.611
3000–5999 beds	1.03	0.51	2.08	0.928
6000–9999 beds	1.18	0.60	2.33	0.633
Over 9999 beds	1.15	0.62	2.11	0.665
Hospital Ownership (ref = Non-Profit)				
For-Profit	0.97	0.72	1.33	0.869
Discharge Quarter (ref = 2020 Q1)				
2020 Q2	0.41	0.12	1.47	0.171
2020 Q3	0.35	0.13	0.98	0.045
2020 Q4	0.55	0.22	1.41	0.213
2021 Q1	0.36	0.14	0.92	0.033
2021 Q2	0.43	0.15	1.21	0.110
2021 Q3	0.93	0.33	2.61	0.885
2021 Q4	0.63	0.23	1.74	0.369

^1^ HR = Hazard ratio. ^2^ CI = confidence interval. ^3^ Race/ethnicity categories are mutually exclusive. NH = Non-Hispanic; AI/AN = American Indian or Alaska Native; NH/PI = Native Hawaiian or Pacific Islander. ^4^ CCI = Charlson Comorbidity Index. ^5^ Critical Access Hospitals (CAHs) are CMS-designated rural facilities with limited inpatient capacity and 24-h emergency care.

**Table A2:** Propensity Score Matching Summary, Covariate Balance, and Matched Cox Model for Black–White Differences in In-Hospital Mortality Among COVID-19-Positive Medicaid Patients, Kentucky, 2020–2021.

**Panel A. Matching Summary**		
	**NH White** ^**1**^	**NH Black** ^**1**^
Unmatched sample (COVID-19-positive inpatients)	6504	1253
Matched 1:1 analytic sample	636	1253
Total matched sample	1889	—
**Panel B. Balance Statistics Before and After Matching (Values shown are proportions in NH White vs. NH Black groups; SD = Absolute Standardized Difference)**		
**Characteristic**	**Before Matching (NH White vs. NH Black, SD ^4^)**	**After Matching (NH White vs. NH Black, SD ^4^)**
Death (in-hospital)	0.064 vs. 0.088, SD = 0.084	0.064 vs. 0.069, SD = 0.015
Age Group (ref = 18–20)		
21–24	0.026 vs. 0.040, SD = 0.077	0.047 vs. 0.040, SD = 0.036
25–29	0.055 vs. 0.083, SD = 0.110	0.097 vs. 0.083, SD = 0.051
30–34	0.066 vs. 0.087, SD = 0.077	0.085 vs. 0.087, SD = 0.007
35–39	0.088 vs. 0.081, SD = 0.023	0.091 vs. 0.081, SD = 0.035
40–44	0.104 vs. 0.117, SD = 0.043	0.113 vs. 0.117, SD = 0.013
45–49	0.117 vs. 0.120, SD = 0.008	0.112 vs. 0.120, SD = 0.025
50–54	0.160 vs. 0.148, SD = 0.034	0.148 vs. 0.148, SD = 0.000
55–59	0.181 vs. 0.152, SD = 0.076	0.151 vs. 0.152, SD = 0.004
60–64	0.188 vs. 0.150, SD = 0.101	0.146 vs. 0.150, SD = 0.011
Sex (ref = Male)		
Female	0.517 vs. 0.516, SD = 0.002	0.498 vs. 0.516, SD = 0.036
CCI Score (ref = 0) ^2^		
1–2	0.318 vs. 0.308, SD = 0.022	0.318 vs. 0.308, SD = 0.021
3–4	0.132 vs. 0.153, SD = 0.062	0.154 vs. 0.153, SD = 0.002
≥5	0.236 vs. 0.247, SD = 0.024	0.237 vs. 0.247, SD = 0.021
Hospital Type (ref = Critical Access) ^3^		
Acute Care	0.463 vs. 0.113, SD = 0.840	0.197 vs. 0.113, SD = 0.234
Hospital Bed Size (ref = <1000 beds)		
1000–2999 beds	0.106 vs. 0.028, SD = 0.315	0.035 vs. 0.028, SD = 0.038
3000–5999 beds	0.154 vs. 0.089, SD = 0.202	0.126 vs. 0.089, SD = 0.120
6000–9999 beds	0.169 vs. 0.161, SD = 0.022	0.223 vs. 0.161, SD = 0.158
Over 9999 beds	0.541 vs. 0.720, SD = 0.378	0.612 vs. 0.720, SD = 0.231
Hospital Ownership (ref = Non-Profit)		
For-Profit	0.106 vs. 0.052, SD = 0.201	0.080 vs. 0.052, SD = 0.114
Discharge Quarter (ref = 2020 Q1)		
2020 Q2	0.026 vs. 0.071, SD = 0.208	0.072 vs. 0.071, SD = 0.005
2020 Q3	0.046 vs. 0.104, SD = 0.223	0.101 vs. 0.104, SD = 0.010
2020 Q4	0.148 vs. 0.171, SD = 0.061	0.176 vs. 0.171, SD = 0.014
2021 Q1	0.160 vs. 0.148, SD = 0.033	0.148 vs. 0.148, SD = 0.000
2021 Q2	0.065 vs. 0.073, SD = 0.035	0.090 vs. 0.073, SD = 0.059
2021 Q3	0.329 vs. 0.241, SD = 0.197	0.217 vs. 0.241, SD = 0.057
2021 Q4	0.221 vs. 0.187, SD = 0.085	0.195 vs. 0.187, SD = 0.021
**Panel C. Propensity Score-Matched Cox Proportional Hazards Model**		
**Group Comparison**	**Adjusted HR (95% CI [Lower—Upper])**	***p*** **-value**
NH Black (ref = NH White)	1.1 (0.69–1.74)	0.692

^1^ NH White = Non-Hispanic White; NH Black = Non-Hispanic Black. ^2^ CCI = Charlson Comorbidity Index. ^3^ Critical Access Hospitals (CAHs) are CMS-designated rural facilities with limited inpatient capacity and 24-h emergency care. ^4^ Standardized differences <0.10 indicate good covariate balance. Note: Values represent the proportion of patients in each covariate category within NH White and NH Black groups. For example, “0.026 vs. 0.040” indicates 2.6% of NH White patients and 4.0% of NH Black patients were ages 21–24. The standardized difference (SD) quantifies balance between groups; SD < 0.10 indicates acceptable balance. Death is the primary outcome and was not used in the matching algorithm. Proportions are shown here for descriptive comparison only. Standardized differences reflect absolute differences in outcome prevalence before and after matching.

**Table A3:** Sensitivity Analysis 1: Cox Models Excluding Length of Stay <1 or >60 Days.

Outcome	Sample (LOS Restricted to 1–60 Days)	Key Exposure/Comparison	Adjusted Effect (95% CI)	*p*-Value
In-hospital mortality (Cox model)	All Medicaid inpatient stays (n = 124,957; 3257 deaths)	COVID-19-positive vs. non-COVID-19 inpatient stays	HR = 2.35 (95% CI: 2.08–2.65)	<0.001
In-hospital mortality (Cox model)	COVID-19-positive inpatients (n = 8371; 689 deaths)	NH Black vs. NH White	HR = 0.90 (95% CI: 0.71–1.14)	0.390

Sensitivity Analysis 1 excluded admissions with length of stay <1 day or >60 days and re-estimated the multivariable Cox models used in the primary analysis. All covariates matched the primary specifications, and robust standard errors were clustered by hospital ID. HR = hazard ratio; CI = confidence interval; LOS = length of stay; NH = non-Hispanic.

**Table A4:** Sensitivity Analysis 2: Models Using Continuous Charlson Comorbidity Index.

Outcome	Sample	Key Exposure/Comparison	Adjusted Effect (95% CI)	*p*-Value
COVID-19 diagnosis among hospitalized patients (logistic regression)	All Medicaid inpatient stays (n = 125,192)	NH Black vs. NH White	OR = 1.40 (95% CI: 1.25–1.58)	<0.001
In-hospital mortality (Cox model)	All Medicaid inpatient stays (n = 125,192)	COVID-19-positive vs. non-COVID-19 inpatient stays	HR = 2.40 (95% CI: 2.12–2.71)	<0.001

Sensitivity Analysis 2 replaced Charlson Comorbidity Index (CCI) categories with the continuous CCI score in both models. All other covariates matched the primary specifications, and robust standard errors were clustered by hospital ID. OR = odds ratio; HR = hazard ratio; CI = confidence interval; CCI = Charlson Comorbidity Index; NH = non-Hispanic.

**Table A5:** Sensitivity Analysis 3: Alternative Propensity Score-Matched Cox Model for COVID-19-Positive NH Black vs. NH White Patients (1:2 Matching).

Outcome	Sample	Matching Specification	Key Exposure/Comparison	Adjusted Effect (95% CI)	*p*-Value
In-hospital mortality (Cox model)	COVID-19-positive NH Black and NH White inpatients (n = 2455; 164 deaths)	1:2 nearest-neighbor propensity score matching (caliper = 0.10) on age group, sex, CCI category, hospital class, bed size, ownership, and discharge quarter	NH Black vs. NH White	HR = 0.86 (95% CI: 0.63–1.17)	0.330

Sensitivity Analysis 3 re-estimated the propensity score-matched Cox model among COVID-19-positive NH Black and NH White patients using 1:2 nearest-neighbor matching with a caliper of 0.10. Matching variables included age group; sex; CCI category; hospital class; bed size; ownership; and discharge quarter. Robust standard errors were clustered by hospital ID. HR = hazard ratio; CI = confidence interval; CCI = Charlson Comorbidity Index; NH = non-Hispanic.
